# Target Capture and Massive Sequencing of Genes Transcribed in *Mytilus galloprovincialis*

**DOI:** 10.1155/2014/538549

**Published:** 2014-06-30

**Authors:** Umberto Rosani, Stefania Domeneghetti, Alberto Pallavicini, Paola Venier

**Affiliations:** ^1^Department of Biology, University of Padua, Via U. Bassi 58/b, 32121 Padua, Italy; ^2^Department of Life Sciences, University of Trieste, Via L. Giorgeri, No. 5, 34121 Trieste, Italy

## Abstract

Next generation sequencing (NGS) allows fast and massive production of both genome and transcriptome sequence datasets. As the genome of the Mediterranean mussel *Mytilus galloprovincialis* is not available at present, we have explored the possibility of reducing the whole genome sequencing efforts by using capture probes coupled with PCR amplification and high-throughput 454-sequencing to enrich selected genomic regions. The enrichment of DNA target sequences was validated by real-time PCR, whereas the efficacy of the applied strategy was evaluated by mapping the 454-output reads against reference transcript data already available for *M. galloprovincialis* and by measuring coverage, SNPs, number of *de novo* sequenced introns, and complete gene sequences. Focusing on a target size of nearly 1.5 Mbp, we obtained a target coverage which allowed the identification of more than 250 complete introns, 10,741 SNPs, and also complete gene sequences. This study confirms the transcriptome-based enrichment of gDNA regions as a good strategy to expand knowledge on specific subsets of genes also in nonmodel organisms.

## 1. Introduction

Genome enrichment methods are efficient ways to reduce sequencing efforts and costs by examining only selected target regions of a given genome [1, 2]. Indeed, the target capture and sequencing approach increases the level of coverage and adds multiplexing options. Various strategies of target enrichment have been employed in many areas of genetic research, from whole exome sequencing [3], sequencing of genes causally linked to diseases [4], and extensive exome resequencing [5] to microbial metagenomics [6] and investigations on ancient DNA [7]. Recently, target enrichment approaches have been applied also to organisms without sequenced genome in order to target the exome for SNP identification or determine gene copy number [8–10]. Before the advent of NGS sequencing, four different methodologies were already available to enrich DNA targets of interest: PCR-based enrichment, microarray- or liquid-based hybridization, restriction enzyme-based enrichment, and physical isolation of mRNA [11]. Among these techniques, in-solution hybrid capture coupled with PCR amplification is one of the most efficient methods for sequencing small- and medium-size targets and it represents a cost-effective procedure in case of low DNA amounts [12, 13].

*Mytilus* spp. are widespread aquaculture bivalves also used as biosensors for coastal water pollution [14].* M. galloprovincialis* has a diploid complement of 28 chromosomes and the haploid DNA content is estimated in 1.38–1.88 Gbp [15]. Its genome is not available and gene sequences are still limited. Nevertheless, mussel gene transcript data are increasing in public databases (19,617 nucleotide sequences, 2,292 proteins, and 319 GEO datasets available at NCBI, May 2014) and support the development of gene-centered studies [16–22]. In order to analyze the molecular variability of transcripts coding for antimicrobial mussel peptides (AMPs) in* M. galloprovincialis*, we have previously made available high-throughput amplicon sequence data [23].

This work aims to increase the current knowledge on the* M. galloprovincialis* genome through the analysis of selectively enriched DNA regions and to assess the feasibility of a small target capture approach in a nonsequenced organism. Toward this end, we have designed a high number of probes on 1,518 mussel transcripts originated by Sanger sequencing [24] and used them to target the related DNA regions by massive 454-sequencing.

## 2. Materials and Methods

### 2.1. DNA and Library Preparation

Genomic DNA (gDNA) was extracted from the foot of one adult mussel using a standard phenol/chloroform method [25]. We assessed the DNA quality on 2% agarose gel with SYBR Safe staining (Invitrogen, Carlsbad, Germany) and DNA concentration with a NanoDrop spectrophotometer (NanoDrop Technologies, Wilmington, USA). The sampled animal was confirmed to be* M. galloprovincialis* by Sanger sequencing of a variable interspecific region of the gene MSLAP [26].

To prepare a single-stranded DNA library, the purified gDNA was fragmented by nebulization and two adapters for GS-FLX sequencing (A 5′-CCATCTCATCCCTGCGTGTCTCCGAC-3′ and B 5′-CCTATCCCCTGTGTGCCTTGGCAGTC-3′) were ligated following the manufacturer's protocol (Roche Life Sciences). Library amount and size were assessed with the NanoDrop 3300 fluorimeter (NanoDrop Technologies) and Bioanalyzer DNA7500 lab-chip (Agilent Technologies, Santa Clara, USA), respectively. The DNA library was then PCR-amplified by using sequencing adapters as forward and reverse primers in order to obtain the quantity necessary for the enrichment procedure.

### 2.2. Probe Design and DNA Enrichment and Sequencing

The* M. galloprovincialis* EST contigs previously catalogued in* Mytibase* were ordered by length. Those longer than 750 bp were selected, so as to design oligonucleotide probes and target the related genomic regions. Shorter sequences representing mussel AMPs were also considered in the probe design, for a total of 1,518 selected contigs covering altogether a genomic target region of 1.35 Mb. RNA probes (120-mers) were designed on the contig sequences, tiled every 60 bp, in order to obtain a final 2× coverage. Biotinylated probes were synthesized by Mycroarray.com (Ann Arbor, USA) with a technology which allows the conversion of a DNA oligonucleotide library into biotinylated RNA baits by* in vitro* transcription. The RNA probe size was accurately checked on RNA6000 lab-chip (Agilent Technologies).

We started the capture-enrichment step from 500 ng of gDNA following manufacturer's instructions [27]. The number of cycles in the postcapture PCR amplification step was set at 15 using a Herculase High Fidelity Taq (Invitrogen) in a Mastercycler Gradient Thermal Cycler (Eppendorf, Hamburg, Germany) as follows: 95°C for 1′, 15 cycles at 95°C for 30′′, 60°C for 30′′, 68°C for 1′, and a final extension step at 68°C for 5′. After purification, the PCR product was subjected to emulsion PCR and 454-sequencing according to standard protocols. Two independent half runs of a PicoTitre plate were performed (BMR Genomics, Padua, Italy).

### 2.3. Enrichment Controls

Four transcripts (3 on-target sequences: MGC05878, MGC00300, and MGC04518; 1 off-target sequence) were evaluated before and after enrichment of the genomic library by RT-PCR analysis. Raw and enriched samples were diluted 10, 50, 100, and 500 times and used as template for RT-PCR, each one with 3 replicates per dilution. PCR was performed in a CFX96 real-time PCR detection system (Bio-Rad) using iQ SYBR Green Supermix (Bio-Rad) with the following thermal profile: 5′ at 95°C, 40 cycles of amplification of 30′′ at 95°C, 20′′ at 56°C, and 30′′ at 72°C. Enrichment ratios were computed with the Ct values resulting from the raw DNA library and the enriched one.

### 2.4. Sequencing Data Analysis

Raw sequencing data are deposited at the NCBI SRA archive under accession number PRJNA246144. The output reads having a minimal Phred quality score (>Q20) [28] were retained for data analysis. Such reads were trimmed for length (>50 nt), quality (<2 Ns), and adaptor sequences. Moreover, duplicated reads and reads mapping on publicly available* Mytilus* microsatellite sequences were excluded. In order to estimate the target coverage, we mapped the trimmed reads on the initially selected EST contigs with the CLC Genomic Workbench, version 5.1 (CLC Bio, Katrinebjerg, Denmark).

All high quality reads, as well as the subsets of on-target and off-target reads, were separately subjected to* de novo* assembling with standard parameters to improve the data analysis (mismatch, insertion, and deletion costs were set at 2∖3∖3, resp.; length fraction and similarity were set at 0.5∖0.8). The contigs resulting from the whole assembly were subjected to SNP discovery and intron-exon analysis. SNPs were prudently considered genuine when coverage was at least 10× and the sequence variation was present at least in 35% of the locally aligned reads. Putative gene structures inferred from the* de novo* contigs were validated using a* M. galloprovincialis* Illumina RNA-seq dataset (SRA ID: PRJNA88481). Briefly, raw Illumina reads were quality trimmed and good quality reads were back mapped on the previously obtained genomic contigs. Evaluation of coverage depth and percentage of covered hits was performed on contigs composed by introns, exons, and the two of them. The latter were used to identify the completely sequenced introns.

### 2.5. Sequence Validation

Six contigs resulting from the genomic assembly (3 intronic sequences, 2 mytilin C regions, and 1 MSLAP gene) were validated by Sanger sequencing. After primer design, PCR was carried out in 50 *μ*L final volume using a Mastercycler Gradient Thermal Cycler set as follows: 95°C for 1′, 35 cycles at 95°C for 30′′, 55°C for 20′′, 68°C for 1′, and a final extension step at 68°C for 5′. Purified PCR products (PureLink PCR purification kit, Invitrogen) were checked by electrophoresis in 2% agarose gel with SYBR Safe staining (Invitrogen) and sequenced in forward and reverse direction (BMR Genomics, Padua, Italy). Primer sequences and other details are available in Supplementary Materials (SM1) (see SM1 in the Supplementary Material available online at http://dx.doi.org/10.1155/2014/538549).

## 3. Results

Using a liquid capture strategy and designing RNA capture probes on the* Mytibase* EST collection [24], we have selected and sequenced a small genome portion of the marine bivalve* M. galloprovincialis*. The target enrichment performed with the* Mybaits* in-solution hybrid capture system (MYcroarray.com) spanned over 1.35 M bases of sequences expressed in the Mediterranean mussel (1,518 contigs targeted by 12,039 RNA probes of 120-mers). Theoretically, our target regions represent 0.1% of the mussel genome, currently estimated at 1.38–1.88 Gbp [15]. For the purpose of comparison, Table 1 reports that the estimated genome size of mollusk species is already sequenced.

We used a medium-size genomic library (average dimension of 680 bp) suitable to the sequencing capacity of the 454 Titanium platform to produce two 454 datasets in parallel (RUN_1 and RUN_2; details are in Table 2 and SM2). Reads of low quality, duplicates, and reads mapping on microsatellite repeats were excluded from the analysis. Clonality of reads, evaluated by counting the identical reads, was around 36%. Therefore, we obtained a total of 626,769 high quality reads (average length: 235 bp) and 354,633 of them could be mapped on the reference set of* Mytibase* transcript sequences, with 1,355 out of 1,518 contigs having at least 1× coverage. Accordingly, the capture efficiency in RUN_1 and RUN_2 was 62% and 52%, respectively.

**Figure 1:**
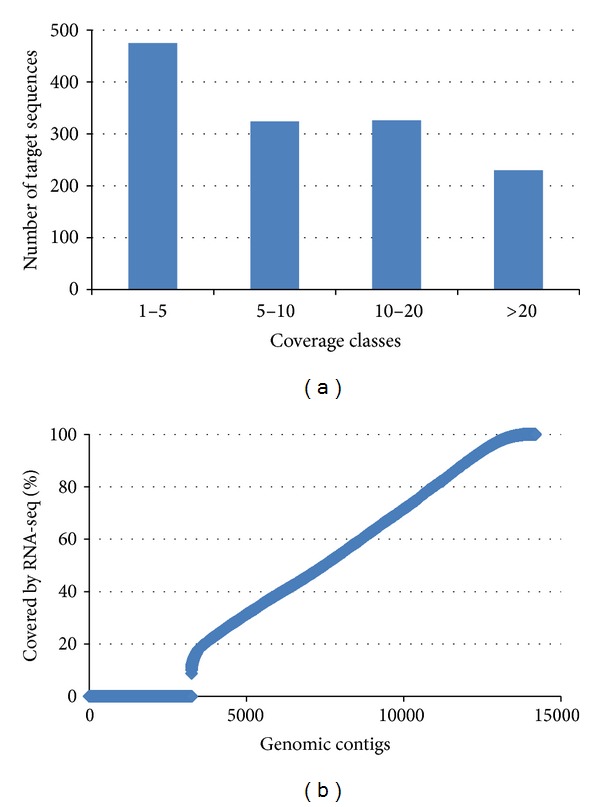
(a) Target coverage distribution. Number of targets per coverage class. (b) Length percentage of the genomic contigs covered by RNA-seq reads.

Most of the 1,355 covered targets had a sequence coverage ranging from 1× to 10× (median value 8×), with about 600 targets displaying more than 5× coverage (Figure 1(a)). We did not observe any positive correlation between target length—directly related to the number of designed probes—and target coverage (SM3).

The sequence coverage observed in the selected cases (3 on-target and 1 off-target sequences) was consistent with the enrichment level measured before sequencing by RT-PCR analysis (Table 3).

*De novo* assembling of the whole sequence read dataset yielded 14,339 contigs, with average length of 511 bp (200–4,217 bp), as summarized in Table 2 and SM4. A first-hit BLAST annotation is reported for the contigs covered with more than 100 reads (SM5).

The reliability of the probe enrichment approach is evident when taking into account the C1q gene family: a total of 1,753 reads were mapped on the 99 gene sequences of the C1q multigene family [34]. In this study, the majority of the mapped reads (96%) matched exclusively with the 28 C1q genes targeted by the designed RNA probes.

**Figure 2:**
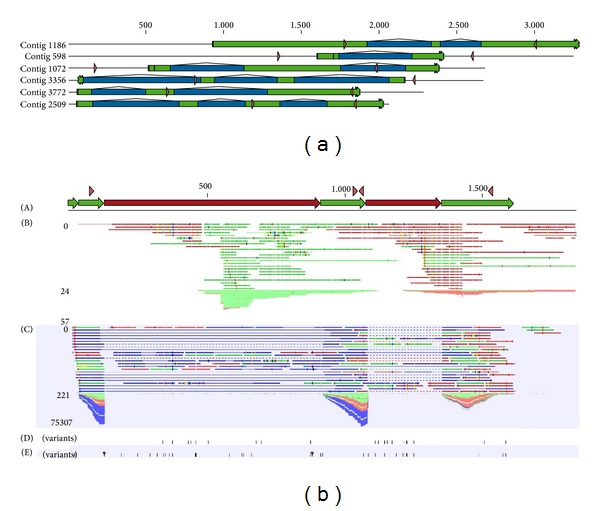
(a) Genomic contigs resulting from* de novo* assembling and evaluated for confirmatory Sanger sequencing. Exon and intron regions are depicted in green and blue, respectively. Pink arrows represent the primer positions. (b) Predicted mytilin C gene: (A) gene structure with exons (green), introns (red), and primer positions (pink); (B) mapping of genomic reads with related coverage graph; (C) mapping of transcriptomic reads with related coverage graph; (D) variant positions detected on genomic sequences; (E) variant positions detected on transcriptomic sequences.

In order to identify gene structures, we mapped our* M. galloprovincialis* read collection (SRA ID: PRJNA88481) on the genomic contigs resulting from this study. More than 29 M reads gave a positive match (Figure 1(b)) and allowed us to predict 4,587 gene regions and identify introns and exons. About 20% of the genomic contigs did not match any RNA-seq read and, either complete or partial, such lack of sequence coverage suggests the presence of introns. Mytilin C (Figure 2) and GADD45 (SM6) exemplify gene structures identified in the assembled genomic contigs. In order to confirm some of the novel introns, we designed primers flanking the intron region and, after amplification, we subjected the PCR products to Sanger sequencing. The resulted sequences, amplified from a different individual mussel, were consistent with the genomic contigs previously assembled (SM1).

With a view to recognizing and counting the fully sequenced introns,* de novo* contigs showing at least 250 bp divergence between the covered length and the total length were blasted against the initial* Mytibase* targets (allowing only one high-scoring segment pair). As a result of retrieving the genomic contigs with more than one hit, we were able to identify 263 fully sequenced introns on a total of 474 contigs (Table 4).

Among other findings, several genomic contigs related to the NF-kB pathway transcripts were identified. For instance, we found the complete gene sequence of MgIRAK-4 composed by one 1,774 bp long exon (GenBank ID KC994891, 533aa; contigs 1408 and 2351) and one MgIkB-a (KF015301, 392aa; contig 5756). Both of these genes include only one exon, a gene structure conserved also in the* C. gigas*,* P. fucata,* and* L. gigantea* genomes. We found the complete MyD88 sequence spread for 2.4 kbp over several genomic contigs and composed by three exons, with the typical DEATH domain completely localized in the first exon and the TIR domain along the remaining ones. A similar gene organization is present in the* C. gigas* (CGI_1002602 and CGI_10013672) and* L. gigantea* (sca_120023) genomes.

**Figure 3:**
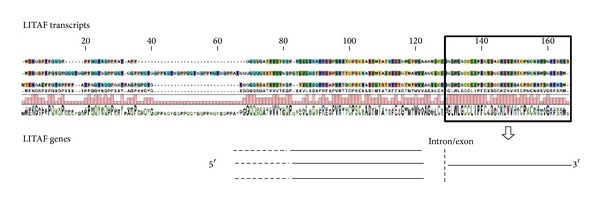
Partial LITAF gene structure. Alignment of the three targeted LITAF transcripts, translated into amino acids. Conservation graph shows the high conservation of the 3′ region, located entirely in one exon. The 5′ region displays a lower conservation level.

With regard to the lipopolysaccharide-induced TNF factor-like sequences (LITAF), seven different transcripts are present in* Mytilus* spp. (GenBank IDs: KF051277 and KF110675-82); this indicates the presence of a multigene LITAF family like in the oyster genome. In this study, the genomic contigs matching the LITAF 4, 5, and 6 transcripts show the same gene organization at the 3′ region. In detail, a common intron/exon junction (Figure 3), with the last 38 highly conserved amino acids entirely localized in one exon, suggests evolutionary diversification by alternative splicing coupled with gene duplication.

The* Mytibase* transcript sequences for the mussel AMPs were included in the list of targets to be enriched, even if their length was less than 750 bp (see M&M). This can explain the low coverage. Regarding mytilin B, the complete 3,125 bp long gene sequence was already available (NCBI ID: AF177540) [16]. Although the presence of at least 3 mytilin transcripts (B, C, and D) has been reported, their gene structures are not yet disclosed. Manual extraction and assembling of the 374 genomic reads aligned on the mytilin targets allowed us to partially reconstruct the gene structure.  Figure 2(b) exemplifies the case of mytilin C.

**Figure 4:**
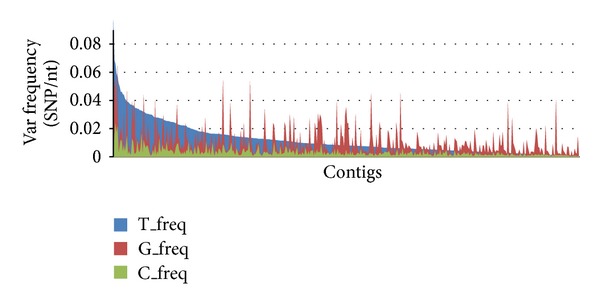
SNP frequency in mussel exons. Predicted frequency of SNPs in exons using transcriptomic data (T, in blue), genomic data (G, in red), and the common SNP dataset (C, in green).

We used both the genomic and transcriptomic reads of* M. galloprovincialis* to evaluate the nucleotide variability per contig. Regarding the genomic reads, the SNP count highlighted 10,741 variable positions in 2,327 out of 14,339 contigs, with an average variation frequency of 0.71% (SNP/nt) and 60% of the SNPs localized on mRNA regions. A higher variation rate was evident by mapping the transcriptomic reads on the same genomic contigs (Table 5). Fewer SNPs are common in the genome and transcriptome datasets: 1,135 SNPs appear in 447 contigs, indicative of 551 transitions and 485 transversions (Figure 4). Among the most variable contigs, only 30% of them display at least a partial functional annotation, suggesting that most of the observed variability is located in intronic sequences.

Despite the low coverage obtained for the AMP-related targets, we could extend the available genomic sequences for some of them (Table 6). As expected, the AMP-related genomic sequences displayed high molecular variability, further confirming the different level of variability previously reported by Mytilin and Myticin gene families [23]. In detail, the intronic nucleotide variants reported by Pallavicini et al. [35] are all confirmed in a single mussel, laying the foundations for investigating the presence of genes coding for nontranslated RNAs or other possible causes of intron conservation in the antimicrobial precursor sequences.

## 4. Discussion

Sequence enrichment strategies take advantage of the NGS high sequencing power and, at the same time, reduce the analytical complexity by focusing on limited portions of a given genome. Nowadays, sequence enrichment protocols can be applied to an increased number of organisms since many genomes of interest have been completely sequenced. Although the genome of* M. galloprovincialis* is not yet sequenced and gene-specific data are scarce, we designed 12 k RNA probes on the Mytibase transcript collection to capture a small portion of the mussel genome by PCR amplification and subsequent 454-sequencing. The* M. galloprovincialis* genome size is remarkable (estimated length: 1.38–1.88 Gbp) and not easily affordable with a random-shotgun approach, due to the number of sequencing runs necessary to obtain an adequate genome coverage and the redundancy expected from repetitive sequences. Focusing on genome regions related to the mussel exome, we defined a small target of study (theoretically, about 0.1% of the nuclear DNA) which could be regarded as preliminary to full genome analysis.

The design of 120-mer probes tiled every 60 nucleotides aimed to ensure, as much as possible, the linear coverage of all targets as reported by Tewhey et al. [12]. Long probes were also chosen to assure a high specificity of the capture reaction, though the presence of introns was expected to interrupt the probe-target matching. Nevertheless, the analysis carried out on the C1q multigene family returned an encouraging result, since only the genes targeted by RNA probes showed a positive read coverage.

We based our analysis on two highly comparable read datasets (RUN1 and RUN2 differ only in their read length) and found a positive correlation between read length and positive matching on target (Table 2, SM2). Overall, 57% of sequenced reads were positively assigned and 89% of targets were covered, consistently with other target capture experiments [3]. On the whole, we observed an average target enrichment of 50×, even though with uneven coverage distribution among targets (SM3). Such uneven distribution could have been influenced by the gene copy number and, more probably, by target redundancy, which might have increased due to the presence of unknown ESTs. It bears remarking that the testing of the four enrichment controls indicated the effectiveness of the enrichment strategy, with comparable results between library enrichment and sequencing coverage.

Data analysis performed on the on-target reads highlighted some procedural constraints, mainly due to the essential differences between the sequenced reads (genomic) and probes designed on transcripts. Moreover, the absence of a mussel genome scaffold makes it difficult to discriminate between completely off-target reads and reads located at 5′ or 3′ of the targets. The* de novo* assembly performed on the whole read dataset was the most effective way to overcome these constraints. Such assembly produced more than 14 k contigs with an average length of 511 bp (range 200–4, 217 bp). In addition, the contig annotation mainly showed sequence similarities to oyster genes, with the percentage of unknown contigs similar to that of other nonsequenced organisms [36, 37].

We estimated the number of intron contigs by counting the contigs without RNA-seq coverage (2.5 k in total, Figure 1(b)). Since 51% of the total contig length was covered by the RNA-seq reads, the remaining 49% (about 3.5 Mbp) may refer to introns or gene transcripts not represented in our RNA-seq data. The presence of other nuclear RNA types has not been analyzed in our work, due to the lack of a complete transcriptome dataset for* M. galloprovincialis*. We then evaluated the number of completely sequenced introns by blasting the genomic contigs against the selected* Mytibase* targets (Table 4). Complete introns were only 3% of the total intron length (i.e., the majority of these introns have still to be completed). Due to the initial choice of a medium-size DNA library which could be completely covered by our 454-sequencing effort, in this study we were not able to fully sequence large introns or to recover regulatory gene elements.

With reference to the resulting data, we found contigs identifying IRAK, IkB, and MyD88 genes, that is, elements belonging to the NFkB pathway recently described in mussels [22, 38] and other important genes (e.g., GADD45, mytilin C, and LITAF). We used these contigs to reconstruct, as much as possible, the gene sequences and compare the gene structure among related species. In the cases of IRAK, GADD45, mytilin C, and IkB, we reconstructed the whole gene. Some of these genes are composed by a single exon, whereas other genes (MyD88, GADD45, and mytilin C) include many exons. In the case of LITAF, we could recover only a part of the gene, and the intercomparison of the several LITAF transcripts in mussels suggests mechanisms for the evolutionary diversification of this multigene family.

We used both the genomic and transcriptomic mussel datasets for SNC analysis, also ranking the contigs on the basis of their sequence diversity. In order to minimize the number of false positives, we applied stringent parameters for SNP calling and removed the reads with identical mapping location. As a matter of fact, variability could be introduced by 454-sequencing [39] and PCR amplification; in particular, errors produced in the early PCR cycles could then be spread in multiple reads with a low possibility to distinguish them from real SNCs. About 60% of the observed variability was located in exome regions and 20% of the SNCs were confirmed by RNA-seq data (Table 5). Furthermore, we analyzed in greater detail the SNPs found in myticin and mytilin genes, known as antimicrobial mussel peptides. Our genomic data support the different sequence diversity previously observed at transcript level [23] and confirm the myticin C as one of the most variable antimicrobials of* M. galloprovincialis,* also at gene level. Moreover, only about 33% of the SNPs present in the analyzed AMP transcripts were detected in the related genomic contigs, probably because the latter refer to the analysis of one individual mussel whereas the RNA-seq reads were produced from pooled mussel samples. Therefore, both transcriptional and posttranscriptional mechanisms are expected to play a role in the production of the observed transcript diversity of mussel AMPs [40, 41].

## 5. Conclusions

This work has exploited the feasibility of a genome-targeted sequencing based only on transcriptomic data of* M. galloprovincialis*. Relying on a continuous and redundant probe design on the expressed mussel sequences, the selected strategy allowed us to improve knowledge on the targeted gene regions, representing a first overview of the genome of the Mediterranean mussel. This work could be further developed by implementing the RNA probe design, library dimension, and total target size. At the same time, whole genome sequencing of* Mytilus spp.* is still underway.

## Supplementary Material

SM1. Confirmatory analysis of six selected genomic amplicons. Contig number and ID; primer pairs, amplicon length, description and related fasta sequences.
SM2. Genomic library before (A) and after (B) amplification (BioAnalyzer High Sensitivity DNA chip, Agilent Technologies). Dimension peak was measured at 690 and 680bp, respectively. Sequencing output data: C) read length distribution, D) GC content, and E) PHRED quality score of the whole 454-sequencing dataset (RUN_1 and RUN_2).
SM3. List of the 1355 target sequences with read coverage. Target length, number of counted reads and average coverage are reported.
SM4. Genomic contigs resulting from de-novo assembling of all 454 genomic reads (fasta format).
SM5. First-hit BLASTX annotation. Sequence data (ID, length and description) and annotation results (hit description and accession ID, e-value and % similarity) are reported for the most covered contigs (> 100 reads).
SM6. Gene structure of contig 2509 (GADD45). A) gene, mRNA and CSD annotation; B) mapping of genomic 454 reads along the contig; C) mapping of trascriptomic Illumina reads along the contig and the coverage graph used to predict the gene structure; D-E) variant positions detected on genome and transcriptome, respectively.

## Figures and Tables

**Table 1:** Genome size of sequenced mollusk species.

Species name	*C*-value (pg)	Length (Gbp)
*Crassostrea gigas* (see Zhang et al. [29])	0.91	1.00
*Lottia gigantean* (see Simakov et al. [30])	0.43	0.35
*Pinctada fucata* (see Takeuchi et al. [31])	/	1.20
*Aplysia californica* (see Broad Institute (US) [32])	1.8/2.0	0.74
*Pecten maximus* (see Biscotti et al. [33])	1.42	1.40

*C*-values (pg) are summarized according to http://www.genomesize.com.

**Table 2:** Sequencing output data and summary of *de novo* assembling results.

Sequencing output	RUN_1		RUN_2		RUN_1 + 2	
Total reads	472,122		473,409		**945,531**	
Total high quality reads	287,362		339,407		**626,769**	
Average length (bp)	380		114		**235**	
On-target reads (number and %)	179,201	62%	175,432	52%	**354,633**	**57%**
Covered targets (number and %)	1,262	83%	1,032	68%	**1,355**	**89%**

*De novo* assembly	On-target reads		Off-target reads		All dataset	

Total contigs	5,547		12,423		**14,339**	
Total assembled reads (number and %)	279,922	79%	347,439	45%	**444,145**	**71%**
Average contig length (bp)	490		476		**511**	
N50 (bp)	557		523		**552**	
N75 (bp)	388		402		**405**	
Longest contig (bp)	2,234		3,538		**4,217**	
Contigs with blast annotation	28%		21%		**44%**	

Total raw and HQ reads, average length (bp), number of mapped reads (on-target), and covered contigs are reported for the subsets (RUN_1, RUN_2) and total sequenced data (RUN_1 + 2).

*De novo* assembly of the reads on-target and off-target and of the whole dataset. Number of resulting contigs and related reads, contig length, quality parameters, longest contig, and percentage of annotated contigs are reported.

**Table 3:** Enrichment fold and coverage of selected transcripts.

ID	Status	Enrichment fold (RT_PCR)	Sequenced reads (NGS)
MGC04518	On-target	32	22
MGC00300	On-target	60	45
MCG05878	On-target	2	7
Target 4	Off-target	−867	/

Enrichment real-time analysis was performed on 3 targets and on 1 not selected transcript (target 4).

Enrichment fold was measured in qRT-PCR by comparing the DNA library before and after enrichment and, subsequently, by reporting the number of reads that mapped uniquely on the targets.

**Table 4:** Fully sequenced introns.

Total contigs with introns	204
Total introns	263
Total intron length (bp)	110,643
Average intron length (bp)	434
Maximum intron length (bp)	1,008
Minimum intron length (bp)	100

**Table 5:** SNP identification in genomic and transcriptomic data of *M. galloprovincialis*.

	Total SNPs	Total contigs with SNPs	SNP frequency (%)	SNPs in exons (%)
Genome	10,741	2,326	0.71	0.58
Transcriptome	13,821	2,057	0.96	0.87
Common	1,135	447	0.31	/

**Table 6:** Overview on *Mytilus* AMPs data.

AMP name	ID NCBI	Reads	Sequence length (NCBI) (bp)	Sequence extension (bp)	SNPs (genomic)	SNPs (transcriptomic)	Common SNPs
Mytilin B	AF177540	271	3,125	0	17	9	3
Mytilin C	/	130	/	1,834	7	23	4
Mytilin D	EU810204	53	/	1,165	8	5	3
Myticin A	/	95	/	1,650	87	54	17
Myticin B	EU088427	72	2,775	0	20	30	14
Myticin C	EU927419	163	1,409	466	61	78	26

Selection of mussel AMPs listed by name, NCBI ID (if present), number of aligned reads, length of public available sequences (bp), sequence elongation (bp), and number of genomic, transcriptomic, and common SNCs.

## Abbreviations

AMP: Antimicrobial peptide
bp: Base pair
EST: Expressed sequence tag
gDNA: Genomic DNA
NGS: Next generation sequencing
nt: Nucleotide
PCR: Polymerase chain reaction
RT-PCR: Real-time PCR
SNP: Single nucleotide polymorphism.
