# Supplementary material for: Target Capture and Massive Sequencing of Genes Transcribed in Mytilus galloprovincialis
Source: Biomed Res Int. 2014 Jun 30;2014:538549. doi: 10.1155/2014/538549 (PMC4101229; doi:10.1155/2014/538549)

Genomic library before (A) and after (B) amplification (BioAnalyzer High Sensitivity DNA chip, Agilent Technologies). Dimension peak was measured at 690 and 680 bp, respectively.

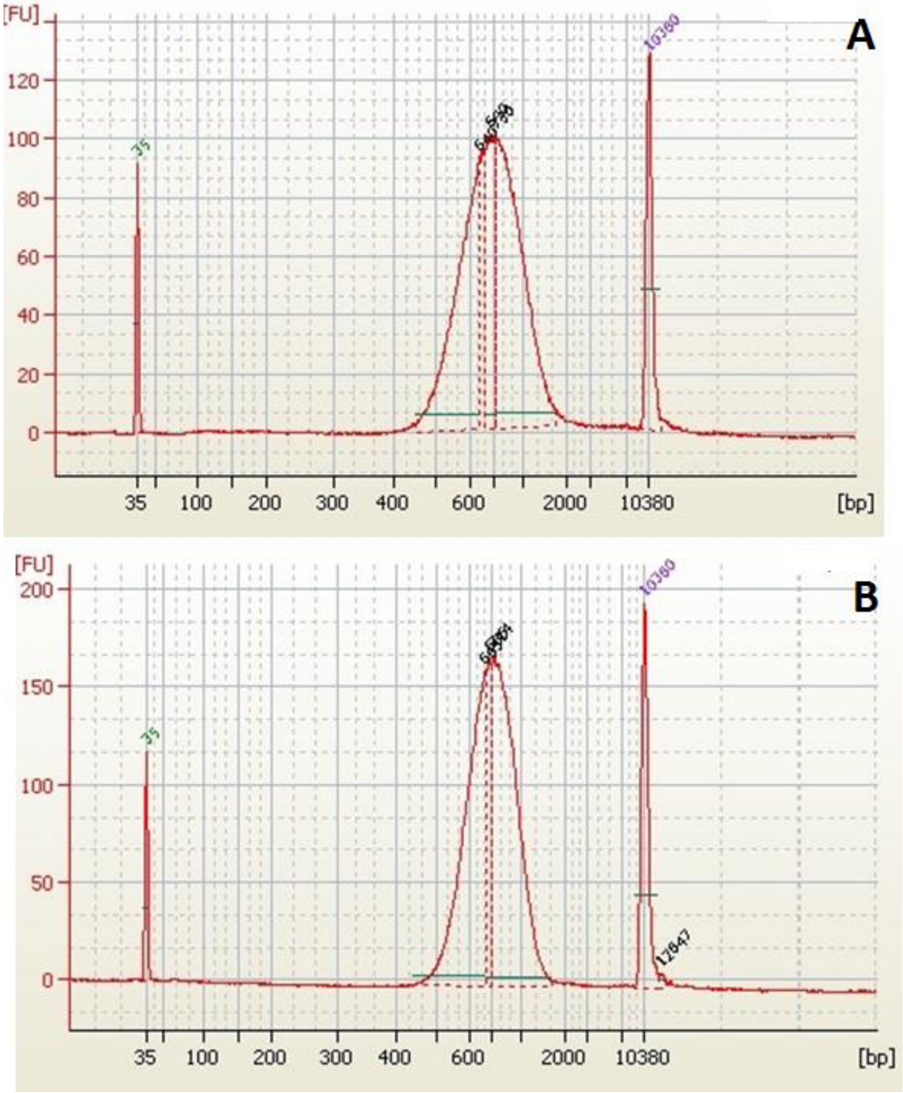

Sequencing output data: C) Read length distribution, D) GC content and E) PHRED quality score of the whole 454-sequencing dataset (RUN_1 and RUN_2).

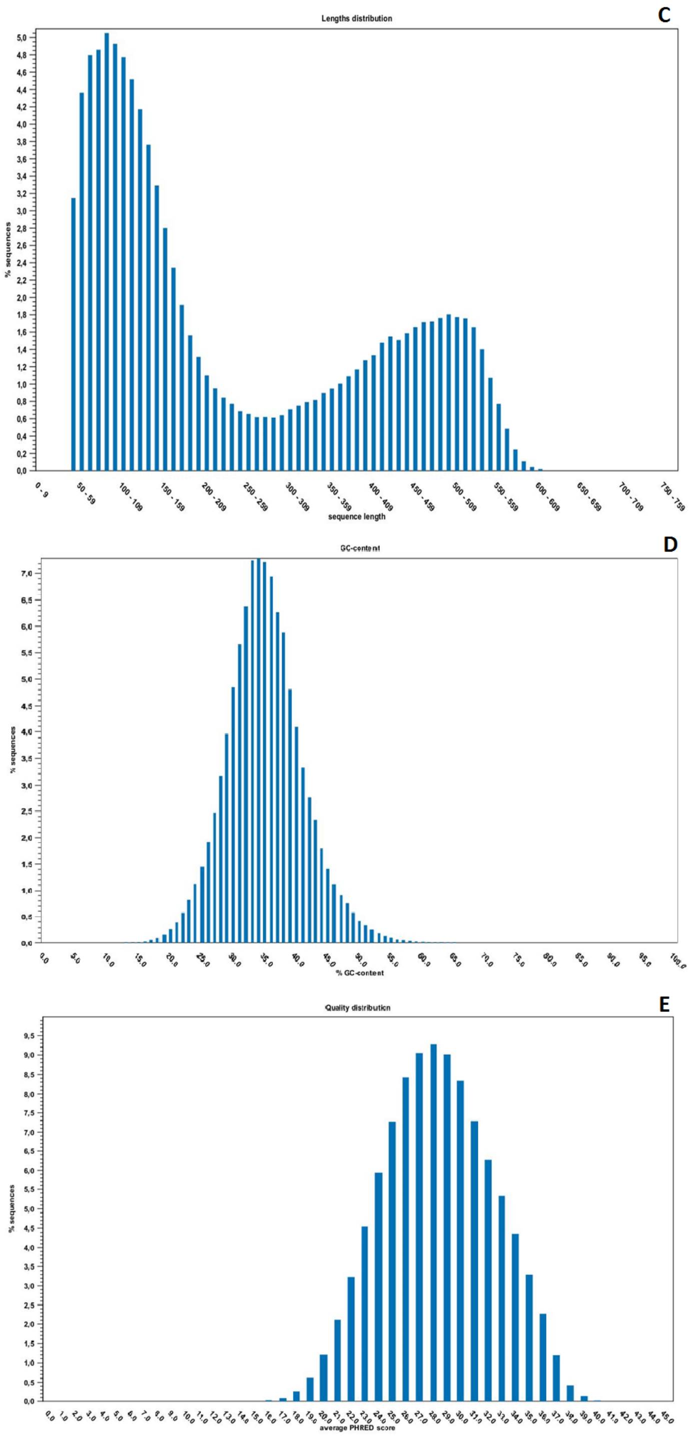

Supplement: Supplementary file 1 — SM1. Confirmatory analysis of six selected genomic amplicons. Contig number and ID; primer pairs, amplicon length, description and related fasta sequences. SM2. Genomic library before (A) and after (B) amplification (BioAnalyzer High Sensitivity DNA chip, Agilent Technologies). Dimension peak was measured at 690 and 680bp, respectively. Sequencing output data: C) read length distribution, D) GC content, and E) PHRED quality score of the whole 454-sequencing dataset (RUN_1 and RUN_2). SM3. List of the 1355 target sequences with read coverage. Target length, number of counted reads and average coverage are reported. SM4. Genomic contigs resulting from de-novo assembling of all 454 genomic reads (fasta format). SM5. First-hit BLASTX annotation. Sequence data (ID, length and description) and annotation results (hit description and accession ID, e-value and % similarity) are reported for the most covered contigs (> 100 reads). SM6. Gene structure of contig 2509 (GADD45). A) gene, mRNA and CSD annotation; B) mapping of genomic 454 reads along the contig; C) mapping of trascriptomic Illumina reads along the contig and the coverage graph used to predict the gene structure; D-E) variant positions detected on genome and transcriptome, respectively. [file 538549.f1.zip › 538549.f1/SM2.pdf]
